# Phase diagram of spiking neural networks

**DOI:** 10.3389/fncom.2015.00019

**Published:** 2015-03-04

**Authors:** Hamed Seyed-allaei

**Affiliations:** School of Cognitive Sciences, Institute for Research in Fundamental Sciences (IPM)Tehran, Iran

**Keywords:** izhikevich spiking neuronal network, phase diagram, recurrent neural network, dynamic range, impulse response, parameter estimation, evolutionary neuroscience

## Abstract

In computer simulations of spiking neural networks, often it is assumed that every two neurons of the network are connected by a probability of 2%, 20% of neurons are inhibitory and 80% are excitatory. These common values are based on experiments, observations, and trials and errors, but here, I take a different perspective, inspired by evolution, I systematically simulate many networks, each with a different set of parameters, and then I try to figure out what makes the common values desirable. I stimulate networks with pulses and then measure their: dynamic range, dominant frequency of population activities, total duration of activities, maximum rate of population and the occurrence time of maximum rate. The results are organized in phase diagram. This phase diagram gives an insight into the space of parameters – excitatory to inhibitory ratio, sparseness of connections and synaptic weights. This phase diagram can be used to decide the parameters of a model. The phase diagrams show that networks which are configured according to the common values, have a good dynamic range in response to an impulse and their dynamic range is robust in respect to synaptic weights, and for some synaptic weights they oscillates in α or β frequencies, independent of external stimuli.

## 1. Introduction

A simulation of neural network consists of model neurons that interact via network parameters; The model is a set of differential equations that describes the behaviors of neurons and synapses, captured by years of electro physiological studies (Nicholls et al., 2012). A model can be as simple as Integrate-and-fire or as realistic as Hodgkin-Huxley (Sterratt et al., 2011). Realistic models generate reliable results, but they are computationally expensive. On the other hand, simple models consume less computer resources, at the cost of loosing details and biological plausibility; therefore, there is a delicate balance between computational feasibility and biological plausibility, Fortunately, there are many reviews and books that compare different models (Izhikevich, 2004; Herz et al., 2006; Sterratt et al., 2011) and neural simulators (Brette et al., 2007). They help us to find a suitable model of a single neuron, set its parameters, and get reliable results in a reasonable time.

No matter how much a neural model is detailed and efficient, A carelessly constructed networks of perfect neural models, is misleading. It only produces wrong biologically plausible results, in a reasonable time. Unfortunately, network parameters are a lot more ambiguous.

There are four essential parameters in a neural network simulation that I investigated in this work - the ratio of excitatory neurons to inhibitory neurons, the topology and sparseness of connections, inhibitory and excitatory synaptic weights. To make it more general and realistic, one may consider the values follow a distribution. This distribution function may change the behavior.

There are experimental works that reads network parameters from nature (Binzegger et al., 2004; Brown and Hestrin, 2009; Lefort et al., 2009; Ko et al., 2011; Perin et al., 2011; Feldmeyer, 2012). For example, *Lefort et al* studied the share of inhibitory neurons in each layer of C2 barrel column of mouse (Lefort et al., 2009). They reported that 100, 16, 10, 8, 17, 17, and 9 percent of layer 1, 2, 3, 4, 5A, 5B, and 6 are inhibitory neurons, and in total, 11 percents of the column is made of inhibitory neurons. They have also studied the synaptic connections of excitatory neurons, they reported neurons of each layers 2, 3, 4, 5A, 5B, and 6 is connected with the probability of 9.3, 18.7, 24.3, 19.1, 7.2, and 2.8 percents to the neurons of the same layer. Moreover, they have studied synaptic weights by measuring unitary Excitatory Post Synaptic Potential (uEPSP), the mean uEPSP for connections inside a layer are 0.64, 0.78, 0.95, 0.66, 0.71, and 0.53 mV for layer 2–6 but the maximum values are much larger, 3.88, 2.76, 7.79, 5.24, 7.16, and 3 mV for the same layers respectively.

The actual values in computer simulations usually are less diverse, In many computational studies, 20% of the neurons are inhibitory, the network topology is a random graph with sparseness of 2% and there are only a couple of fixed synaptic weights for inhibitory and excitatory neurons. Despite their simplicity, they observe many interesting phenomena like oscillations, synchrony, modulations (Song et al., 2000; Brette et al., 2007; Goodman and Brette, 2008; Buice and Cowan, 2009; Akam and Kullmann, 2010; Mejias and Longtin, 2012; Augustin et al., 2013) and even psychological disorders (Bakhtiari et al., 2012). There are also many works that show the results are not actually sensitive to the parameters (Prinz et al., 2004; Marder et al., 2007; Marder, 2011; Marder and Taylor, 2011; Gutierrez et al., 2013).

Why do this parameters appear in nature? What are their evolutionary advantages? How much is the result of a simulation robust in respect to the parameters? How does one decide about the network parameters in a simulation? Which other parameters might give the same results? These are few question that come to mind, while dealing with properties of neural networks, and they could be easily answered by looking at a phase diagram of neural networks, that shows the behavior of a network in respect to its parameters. Like the phase diagram of water, tells us about the properties of water in different pressures and temperatures. But such a phase diagram does not exist for spiking neural networks, yet. First, because of the large amount of computation needed and second, because of the ambiguity of the concept of phase or state in neural networks.

To answer these question, I could use sophisticated evolutionary algorithms, like the study of evolution of inteligence by McNally et al. (2012), however, I used a simple brute force search in the space of parameters, so I can also generate a phase diagram, similar to the work of Roy et al. (2013).

## 2. Method

I did a brute force search in the space of parameters, by simulating a population of 1000 neurons, let say a part of a single layer of cortex, that receives excitatory signals from previous layer of cortex or an external stimuli, and then sends excitatory signals to the next layer. There are lateral connections among all types of neurons (excitatory and inhibitory) in a single layer, but only excitatory neurons interconnects different layers. I stimulated this network and watched its response. This was repeated over a wide range of parameters. At the end I calculated fitness of each set of parameters and then investigated the overlap of the best parameters according to my simulation with the nature's choices (or computational neuroscientists' choices).

To do any sort of evolutionary analysis, first we need to agree on something to optimize –a fitness function, it can be the network capacity to learn and store information (Barbour et al., 2007), or it can be the speed and the quality of transmitted signals (Chklovskii et al., 2002). But here, to define the fitness function, I remind you of three obvious evolutionary facts:

1. High *Dynamic Range* is evolutionary favored, an animal that can see during days and nights, has a higher survival chance than an animal which can see only during days.
2. Small *Just-Noticeable Difference* is evolutionary favored, an animal which can discriminate more gray levels or colors, has a better chance to find its preys and foods.
3. Low *Energy Consumption* is also favored, an energy efficient animal, consumes less food and therefore has a better chance to survive (Niven and Laughlin, 2008).

Dynamic Range is the ratio of the largest possible value of stimuli to its smallest perceivable value. Just-Noticeable Difference is the smallest detectable difference between two sensory stimuli, which is often roughly proportional to the magnitude of the stimulus. The ratio of Just-Noticeable Difference to the magnitude of the stimulus is known as Weber constant (Dayan and Abbott, 2001).

**Figure 1:**
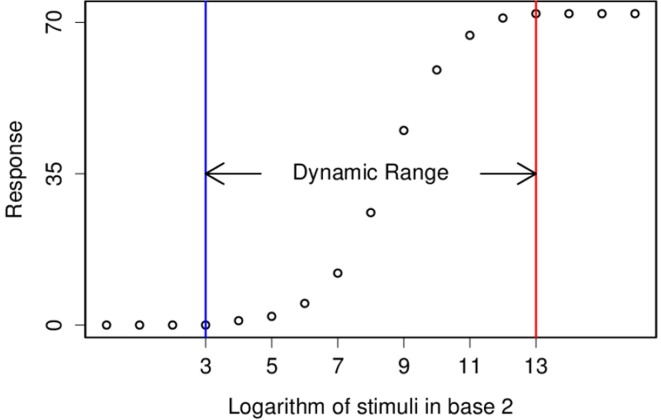
**This figure demonstrates a typical tuning curve and its dynamic range**. Two consecutive stimuli are different by a factor of 2, also known as one *stop*.

Both Dynamic Range and Just-Noticeable are well presented in logarithmic scale, but instead of log_10_ and dB, from now on, I will use log_2_ and *stop* which are common in Optics and Photography (Figure 1).

Here we are left with a neural network and three concepts to play with—Dynamic Range, Just-Noticeable Different and Energy Consumption. To keep it simple, I only measure Dynamic Range while there are upper bounds for the other two. I require Just-Noticeable Different to be smaller than one *stop*, thus, the calculated Dynamic Range is a lower bound for the real Dynamic Range, and I require the Energy Consumption to be zero in absence of stimulus, which puts an upper bound for the Energy Consumption. Moreover, energy consumption can be a determinant factor when all other conditions are equal, when there are two sets of parameters with about the same Dynamic Range, the one which consumed less energy will be favored, and here, the measure of energy consumption is the number generated spikes (Attwell and Laughlin, 2001).

I stimulated the network with a set of stimuli that were equally spaced in the logarithmic scale, separated by 1 stop (× 2) intervals, similar to Figure 1. I ran the simulation for a given time 1024 ms and then I calculated Dynamic Range over the range that Just-Noticeable Difference was smaller than 1 stop.

To be precise, I used a sequence of *n* stimuli plus the resting state (no stimulus), so the sequence includes *n* + 1 members *s* ϵ (*s*_0_, *s*_1_, *s*_2_, …, *s_n_*). Except *s*_0_, the resting state, the difference between *i*-th and (*i* + 1)-th stimulus is one stop: *s*_*i* + 1_ = 2*s_i_. Each stimulus *s_i_* evokes a response *r*(*s_i_*) ϵ *R*, where *R* = (*r*(*s*_0_), *r*(*s*_1_), *r*(*s*_2_), …, *r*(*s*_n_)). Now, the Dynamic Range is simply the number of elements in the largest subsequence of *R*′ ⊂ *R* that fulfills the expectations,

1. For any *r*(*s_i_*), *r*(*s_j_*) ϵ *R*′, we should have *s_i_* < *s_j_* ⇒ *r*(*s_i_*) < *r*(*s_j_*). It means that *R*′ is strictly monotonic and it satisfies the constrain on the Just-Noticeable difference.
2. For any *s_i_* ϵ *R*′, the network must return to resting state in acceptable time. This satisfy the Energy Consumption constrains.

For example, if stimuli (0, 1, 2, 4, 8, 16) leads to the responses (0, 0, 2, 3, 4, 4) then the subsequence (0, 2, 3, 4) ⊂ *R* is the largest subsequence of *R* which meets all the conditions; Therefore, the dynamic range is 4. In this way the minumum dynamic range can be 1 and its maximum can reach the total number of stimuli.

In this study I assumed response *r*(s) as the maximum firing rate of population of excitatory neurons in response to the stimulus *s*. But the paradigm of this study is general and can be applied to any neural coding schemes.

I stimulated some of the neurons with an impulse, I forced *s_i_* excitatory neurons to fire once at time *t* = 0, then I monitored the propagation of the impulse. Here the number of excited neurons represents the magnitude of the stimuli *s_i_*. This is very similar to Optogenetics stimulation, in which, a laser pulse excites a specific population of neurons (Peron and Svoboda, 2011; Toettcher et al., 2011; Lim et al., 2012; Liu et al., 2012).

I used a *simple compartment Izhikevic model* (Izhikevich, 2003). It can imitate the behavior of Hodgkin-Huxley model, and its computational costs is comparable to the Integrate-and-fire model (Izhikevich, 2004). It is based on a system two-dimensional differential equations:

(1) $$\frac{dv}{dt}=0.04v^{2}+5v+140-u+I$$

(2) $$\frac{dw}{dt}=a(bv-u)$$

and the after spike equation of:

$$\text{if }v>30\text{mV, then}\{\begin{array}{l} v\leftarrow c\\ u\leftarrow u+d \end{array}$$

all units are in *mV* and *ms*. Here *v* is the membrane potential, *I* is the input current and *u* is a slow variable that imitate the effect of slow moving ions in the cell, like Calcium ions. With a proper values of parameters *a, b, c*, and *d*, it can model regular spiking neurons, fast spiking neurons and it includes adaptation. In our simulaton excitatory neurons are assumed to be regular spiking, inhibitory neurons are assumed to be fast spiking. The values of parameters and the initial values of *u* and *v* are set as follow:

**Table:** 

	**a**	**b**	**c**	**d**	**u**	**v**
Regular spiking	0.02	0.2	−65.0	8.0	−14	−70
Fast spiking	0.10	0.2	−65.0	2.0	−14	−70

My network has a random graph topology (Erds and Rényi, 1960). In a random graph, every two neurons are connected by probability *p*, called sparseness. This is one of the simplest topology for a network or graph.

I simulated networks of *N* = 1000 neurons with

$$N_{I}\in (100,\;200,\;300,\;400,\;500)$$

inhibitory neurons. The neurons were connected by a random graph topology, with the sparseness

p∈(0.01, 0.02, 0.04, 0.08, 0.16).

On the other hands, the synaptic weights are chosen randomly, but in a way to cover the region of interest in the *w_e_*, *w_i_* plane. In this way, it is always possible to add new points later to improve results. The new points could be just another pair of connection weights(*w_e_*, *w_i_*) on the same random netwrok, or they could be synaptic weights of a whole new realiziation.

**Figure 2:**
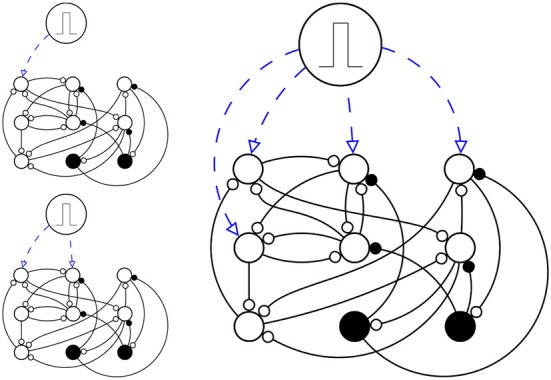
**A simplified schema of simulation setup**. Here there are just *N* = 9 neurons (*N* = 1000 in the actual simulation), in which *N_e_* = 7 are excitatory neurons (circles) and *N_i_* = 2 are inhibitory neurons (disks). The network is stimulated three times, each time *s* ϵ (1, 2, 4) neurons are stimulated (up left, down left and right respectively), with a pulse, in a way that they fire once at time *t* = 0 and then they are like any other neuron in the network.

I stimulated each network 10 times,

s∈(0, 1, 2, 4, 8, 16, 32, 64, 128, 256)

so in each trial, *s* neurons fire at *t* = 0. Then I watched the network for 1024 ms. At the end, I calculated Dynamic Range of the network. I repeated the whole process for few realization of random networks and and then the median of all data in each hexagonal is calculated and displayed (Figure 2).

The whole process needs a great amount of computational power. That is the reason I used *NeMo* (http://nemosim.sourceforge.net/), a neural simulator software that runs on GPU (Fidjeland et al., 2009; Fidjeland and Shanahan, 2010), but for the pilot study, I used *Brian* (http://briansimulator.org/) (Goodman and Brette, 2008). Both of them are open source and available on their websites.

## 3. Results

**Figure 3:**
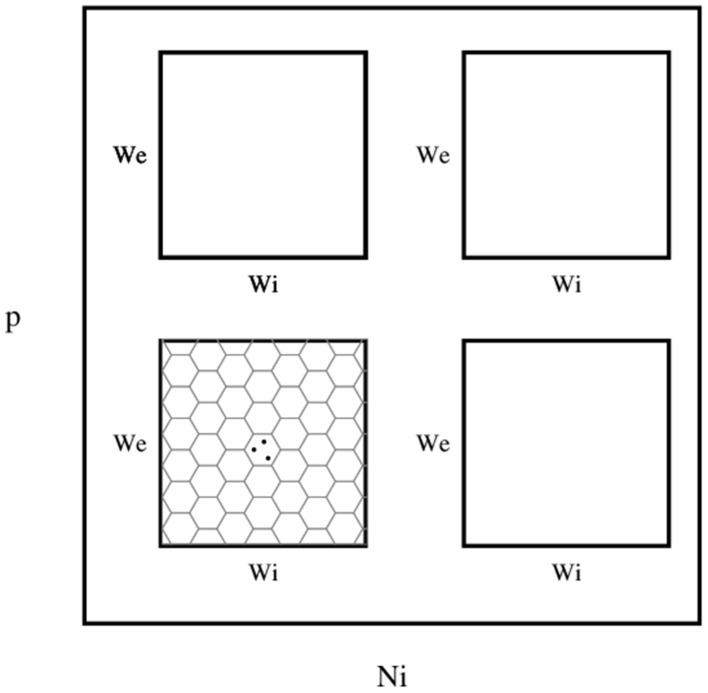
**The layout of phase diagram, (Figure 4 and others), Here, *p* is the sparsness of connections, *N_i_* is the number of inhibitory neurons, *w_e_* is the weight of excitatory synapses (a positive number) and *w_i_* is the weight of inhibitory synapses (a negative number)**. The plane of *w_e_* and *w_i_* is devided into hexagonal bins. The color of each hexagon is defined by the aggregated value of data points inside that hexagon. For aggregation, unless spesified, median value of points are used. If a hexagon is empty, then its color is white.

**Figure 4:**
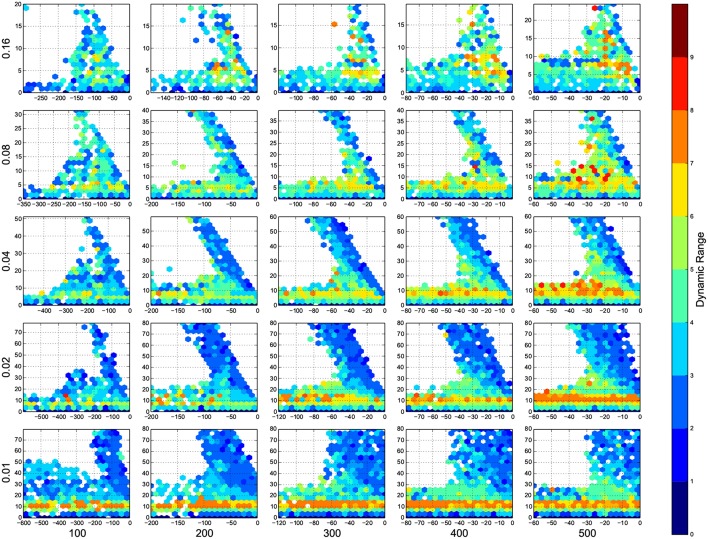
**The phase diagram of dynamic ranges, dark red means high dynamic range (good), white means there was not any simulation data at that bin, all the data were unreliable, or the the network did not come back to its resting state before 100 ms of simulation**. The information are arranged according to the template of Figure 3.

**Figure 5:**
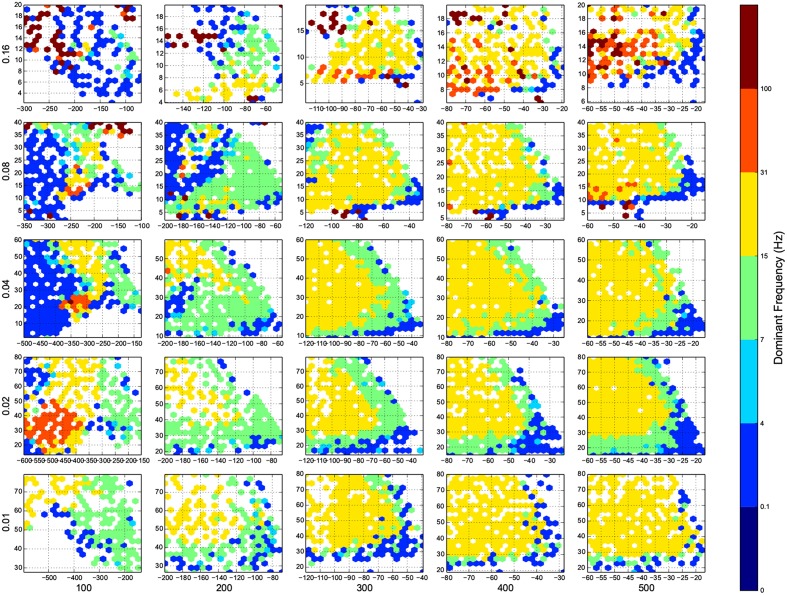
**The phase diagram of oscillations, the dominant frequency of population rate of the network acording to FFT**. Colors are associated to brain waves: δ (0.1-4 Hz), θ (4-7 Hz), α (7-15 Hz), β (15-31 Hz), and γ(31-100 Hz), and color of a bin shows the median of all trials in that bin. Here white means there was not any simulation data at that bin, all the data were unreliable (they fired at the maximum possible population firing rate), or the network did not show any activity after 100 ms of simulation. The information are arranged according to the template of Figure 3.

**Figure 6:**
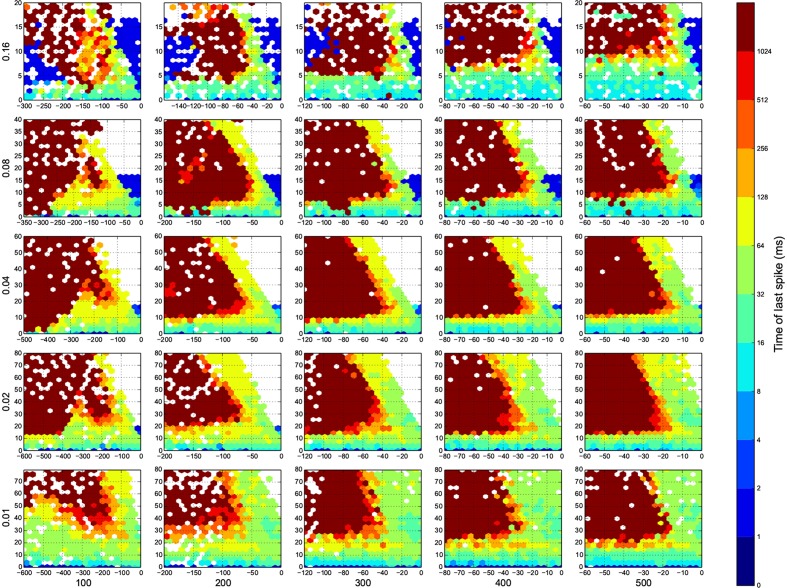
**The time of last spike in each trial**. Dark red means the simulation ended before the network returns to its resting state. Here white means there was not any simulation data at that bin, or all the data were unreliable. The information are arranged according to the template of Figure 3.

**Figure 7:**
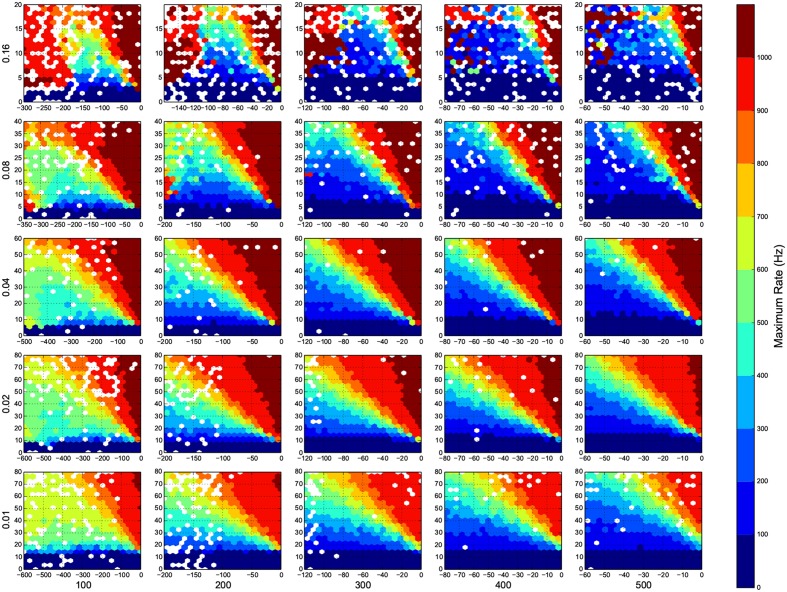
**Maximum firing rate in each trial**. Here white means there was not any simulation data at that bin. The information are arranged according to the template of Figure 3.

**Figure 8:**
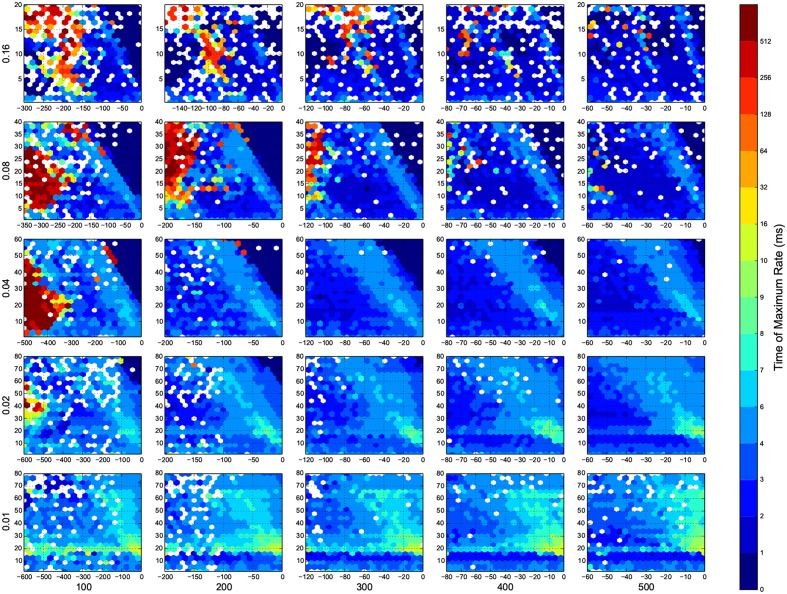
**The time that maximum firing rate happens in each trial**. Here white means there was not any simulation data at that bin. The information are arranged according to the template of Figure 3.

I wanted to present the results as a function of 4 other parameters, for that I needed to map a 4-D space to a 2-D space. Here I used the template in Figure 3, to present the dynamic ranges in Figure 4, oscillations in Figure 5, diuration of activities in Figure 6, maximum achived firing rate in each trial in Figure 7 and the time of achived maximum rate in Figure 8 First I made the plots of dynamic range in the space of synaptic weights – *w_e_* and *w_i_*. Then I put these plots next to each other according to sparseness of connections *p* and number of inhibitory neurons *N_i_*.

## 4. Discussion

In Figure 4, top right corner of each figure is empty (white). This is where the excitatory neurons dominates and the network riches its maximum firing rate, this region is visible in Figure 7 as the darkest shade of red. The lesft side of each graph, where the inhibitory subnetwork dominates, is also empty, but this time because networks did not return to resting states. More detail about the length of activity demonstrated in Figure 6.

A narrow band seperate this two regins, on this band, the inhibitory and excitatory network are balancing out each other. As we increase the number of connections, this band gets narrower, the delicate balance can be easily disturbed. The dynamic ranges on this band is low or moderate.

At the bottom of each graph, there is another region with valid data points. In this region, excitatory sub networks are not strong enough to saturate the system, so we have a good dynamic range regardless of the inhibitory synaptic weights. Nevertheles, stronger inhibitory sub networks improves the dynamic range.

**Figure 9:**
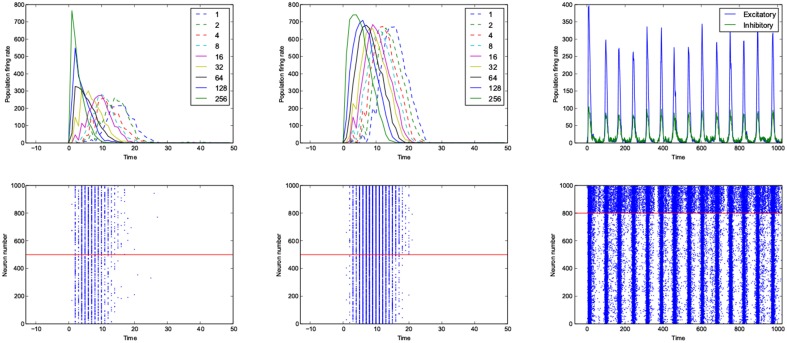
**An example of fast respoding network with a good dynamic range (left), poor dynamic range (middle) and an example of oscillatory network (right)**. Population rates as a function of time (top). The spiking avtivity of neurons (bottom). The red line seperates excitatory neurons (below the line) from inhibitory neurons (above the line).

For the calculation of dynamic range, only trials who returned to resting state have been used. But in many other trials the network sustained its activity upto the very last miliscond of simulation (Figure 6). These networks shows interesting oscillatory behaviures, that can be seen in Figures 5, 9. The oscillations are mostly α and β waves, with fewer case of γ waves. There are also δ and θ waves, but the simulation time of 1 s may not be enough to study these waves.

The networks are stimulated with only a pulse, and then they are left alone, even though there is not any spontaneous activity implemented in the model, yet, the networks show sustained oscillations.

The oscillation are mostly β-waves, and they happen in the region of dominating inhibitory sub networks. As we get to the balanced inhibitory-excitatory band, the oscillations become α-waves.

It seems that the results are not very sensitive to the parameters, We can have good dynamic range over some value of parameters, and we can have neural oscillators in different settings. These finding agrees with Prinz et al. (2004); Marder et al. (2007); Marder and Taylor (2011); Marder (2011); Gutierrez et al. (2013).

The results of this paper, specially Figures 4, 5, serves as a starting point to decide about parameters in a neural simulation, and they may help to find the networks with desired behavior.

This work by no mean is complete, I only studied the effect of four parameters, in just one model, over dynamic range and oscillations. Many other parameters could be included, like synaptic delay and distribution of weights or connections. Also it is interesting to know if my results holds for simpler models, like Integrate-and-fire.

At the end I suggest that any numerical simulation of neural network should be accompanied with such a phase diagram, it demonstrate the robustness of the results in respect to the parameters. One good example is the the work of Roy et al. (2013), where they made such diagram to compare experimental data of orientation selectivity index of mouse primary virtual cortex to their model.

### Conflict of interest statement

The author declares that the research was conducted in the absence of any commercial or financial relationships that could be construed as a potential conflict of interest.

## References

[B1] Akam T. Kullmann D. M. (2010). Oscillations and filtering networks support flexible routing of information. Neuron 67, 308–320. 10.1016/j.neuron.2010.06.019 20670837 PMC3125699

[B2] Attwell D. Laughlin S. B. (2001). An energy budget for signaling in the grey matter of the brain. J. Cereb. Blood Flow Metab. 21, 1133–1145. 10.1097/00004647-200110000-00001 11598490

[B3] Augustin M. Ladenbauer J. Obermayer K. (2013). How adaptation shapes spike rate oscillations in recurrent neuronal networks. Front. Comput. Neurosci. 7:9 10.3389/fncom.2013.00009 PMC3583173 23450654

[B4] Bakhtiari R. Mohammadi Sephavand N. Nili Ahmadabadi M. Nadjar Araabi B. Esteky H. (2012). Computational model of excitatory/inhibitory ratio imbalance role in attention deficit disorders. J. Comput. Neurosci. 33, 389–404. 10.1007/s10827-012-0391-y 22566142

[B5] Barbour B. Brunel N. Hakim V. Nadal J.-P. (2007). What can we learn from synaptic weight distributions? Trends Neurosci. 30, 622–629. 10.1016/j.tins.2007.09.005 17983670

[B6] Binzegger T. Douglas R. J. Martin K. A. C. (2004). A quantitative map of the circuit of cat primary visual cortex. J. Neurosci. 24, 8441–8453. 10.1523/JNEUROSCI.1400-04.2004 15456817 PMC6729898

[B7] Brette R. Rudolph M. Carnevale T. Hines M. Beeman D. Bower J. M.. (2007). Simulation of networks of spiking neurons: a review of tools and strategies. J. Comput. Neurosci. 23, 349–398. 10.1007/s10827-007-0038-6 17629781 PMC2638500

[B8] Brown S. P. Hestrin S. (2009). Intracortical circuits of pyramidal neurons reflect their long-range axonal targets. Nature 457, 1133–1136. 10.1038/nature07658 19151698 PMC2727746

[B9] Buice M. A. Cowan J. D. (2009). Statistical mechanics of the neocortex. Prog. Biophys. Mol. Biol. 99, 53–86. 10.1016/j.pbiomolbio.2009.07.003 19695282

[B10] Chklovskii D. Schikorski T. Stevens C. (2002). Wiring optimization in cortical circuits. Neuron 34, 341–347. 10.1016/S0896-6273(02)00679-7 11988166

[B11] Dayan P. Abbott L. (2001). Theoretical Neuroscience: Computational and Mathematical Modeling of Neural Systems. Cambridge, MA: MIT Press.

[B12] Erds P. Rényi A. (1960). On the evolution of random graphs. Publ. Math. Inst. Hungar. Acad. Sci. 5, 17–61.

[B13] Feldmeyer D. (2012). Excitatory neuronal connectivity in the barrel cortex. Front. Neuroanat. 6:24. 10.3389/fnana.2012.00024 22798946 PMC3394394

[B14] Fidjeland A. K. Shanahan M. P. (2010). Accelerated simulation of spiking neural networks using GPUs, in The 2010 International Joint Conference on Neural Networks (IJCNN) (Barcelona), 1–8.

[B15] Fidjeland A. K. Roesch E. B. Shanahan M. P. Luk W. (2009). NeMo: a platform for neural modelling of spiking neurons using GPUs, in 2009 20th IEEE International Conference on Application-specific Systems, Architectures and Processors (Boston, MA), 137–144.

[B16] Goodman D. Brette R. (2008). Brian: a simulator for spiking neural networks in python. Front. Neuroinform. 2:5. 10.3389/neuro.11.005.2008 19115011 PMC2605403

[B17] Gutierrez G. J. O'Leary T. Marder E. (2013). Multiple mechanisms switch an electrically coupled, synaptically inhibited Neuron between competing rhythmic oscillators. Neuron 77, 845–858 10.1016/j.neuron.2013.01.016 23473315 PMC3664401

[B18] Herz A. V. M. Gollisch T. Machens C. K. Jaeger D. (2006). Modeling single-neuron dynamics and computations: a balance of detail and abstraction. Science 314, 80–85. 10.1126/science.1127240 17023649

[B19] Izhikevich E. M. (2003). Simple model of spiking Neurons. IEEE Trans. Neural Netw. 14, 1569–1572. 10.1109/TNN.2003.820440 18244602

[B20] Izhikevich E. M. (2004). Which model to use for cortical spiking neurons? IEEE Trans. Neural Netw. 15, 1063–1070. 10.1109/TNN.2004.832719 15484883

[B21] Ko H. Hofer S. B. Pichler B. Buchanan K. A. Sjöström P. J. Mrsic-Flogel T. D. (2011). Functional specificity of local synaptic connections in neocortical networks. Nature 473, 87–91 10.1038/nature09880 21478872 PMC3089591

[B22] Lefort S. Tomm C. Floyd Sarria J.-C. Petersen C. C. H. (2009). The excitatory neuronal network of the C2 barrel column in mouse primary somatosensory cortex. Neuron 61, 301–316. 10.1016/j.neuron.2008.12.020 19186171

[B23] Lim D. H. Mohajerani M. H. Ledue J. Boyd J. Chen S. Murphy T. H. (2012). *In vivo* large-scale cortical mapping using channelrhodopsin-2 stimulation in transgenic mice reveals asymmetric and reciprocal relationships between cortical areas. Front. Neural Circuits 6:11 10.3389/fncir.2012.00011 PMC3304170 22435052

[B24] Liu X. Ramirez S. Pang P. T. Puryear C. B. Govindarajan A. Deisseroth K.. (2012). Optogenetic stimulation of a hippocampal engram activates fear memory recall. Nature 484, 381–385. 10.1038/nature11028 22441246 PMC3331914

[B25] Marder E. Taylor A. L. (2011). Multiple models to capture the variability in biological neurons and networks. Nat. Neurosci. 14, 133–138. 10.1038/nn.2735 21270780 PMC3686573

[B26] Marder E. Tobin A.-E. Grashow R. (2007). How tightly tuned are network parameters? Insight from computational and experimental studies in small rhythmic motor networks. Prog. Brain Res. 165, 193–200. 10.1016/S0079-6123(06)65012-7 17925247

[B25a] Marder E. (2011). Variability, compensation, and modulation in neurons and circuits. Proc. Natl. Acad. Sci. U.S.A. 108(Suppl.), 15542–15548. 10.1073/pnas.1010674108 21383190 PMC3176600

[B28] McNally L. Brown S. P. Jackson A. L. (2012). Cooperation and the evolution of intelligence. Proc. R. Soc. B Biol. Sci. 279, 3027–3034 10.1098/rspb.2012.0206 PMC3385471 22496188

[B29] Mejias J. Longtin A. (2012). Optimal heterogeneity for coding in spiking neural networks. Phys. Rev. Lett. 108, 1–5. 10.1103/PhysRevLett.108.228102 23003656

[B30] Nicholls J. G. Martin A. R. Fuchs P. A. Brown D. A. Diamond M. E. Weisblat D. A. (2012). From Neuron to Brain, 5th Edn. Sunderland, MA: Sinauer Associates.

[B31] Niven J. E. Laughlin S. B. (2008). Energy limitation as a selective pressure on the evolution of sensory systems. J. Exp. Biol. 211(Pt 11), 1792–1804. 10.1242/jeb.017574 18490395

[B32] Perin R. Berger T. K. Markram H. (2011). A synaptic organizing principle for cortical neuronal groups. Proc. Natl. Acad. Sci. U.S.A. 108, 5419–5424. 10.1073/pnas.1016051108 21383177 PMC3069183

[B33] Peron S. Svoboda K. (2011). From cudgel to scalpel: toward precise neural control with optogenetics. Nat. Methods 8, 30–34. 10.1038/nmeth.f.325 21191369

[B34] Prinz A. A. Bucher D. Marder E. (2004). Similar network activity from disparate circuit parameters. Nat. Neurosci. 7, 1345–1352. 10.1038/nn1352 15558066

[B35] Roy D. Tjandra Y. Mergenthaler K. Petravicz J. Runyan C. A. Wilson N. R. (2013). Afferent Specificity, Feature Specific Connectivity Influence Orientation Selectivity: A Computational Study in Mouse Primary Visual Cortex. Available online at: http://arxiv.org/abs/1301.0996

[B36] Song S. Miller K. D. Abbott L. F. (2000). Competitive hebbian learning through spike-timing-dependent synaptic plasticity. Nat. Neurosci. 3, 919–926. 10.1038/78829 10966623

[B37] Sterratt D. Graham B. Gillies A. (2011). Principles of Computational Modelling in Neuroscience. Cambridge: Cambridge University Press

[B38] Toettcher J. E. Voigt C. A. Weiner O. D. Lim W. A. (2011). The promise of optogenetics in cell biology: interrogating molecular circuits in space and time. Nat. Methods 8, 35–38 10.1038/nmeth.f.326 21191370 PMC3024327

