# Supplementary material for: Phase diagram of spiking neural networks
Source: Front Comput Neurosci. 2015 Mar 4;9:19. doi: 10.3389/fncom.2015.00019 (PMC4349167; doi:10.3389/fncom.2015.00019)
Supplement: Supplementary file 1 [file Presentation1.PDF]

phase-diagram-demo

August 16, 2014

1 Phase Diagram of Spiking Neural Networks (Demo)

This is the example of the code used to generate the phase diagrams. It uses NeMo as the neural simulator engine.

```
In [1]: import networkx as nx  # we need this to make a random graph.
        import numpy as np
        import time
        import nemo  # The neural simulation backend, http://nemosim.sourceforge.net/
        import matplotlib.pyplot as plt  # to make some pretty plots.
        import pandas as pd  # to store data.
        import os.path
        from IPython.display import Image

        conf = nemo.Configuration()

        # unmark this if you want to use CUDA backend.
        #conf.set_cuda_backend(-1)
```

1.1 Parameters

Here we set the parameters of the simulation: number of simulations, Number of neurons, sparseness of connections and etc. There are inline description for each parameters.

```
In [2]: filename="demo.csv"  # filename to read/save data.

        points = 100  # number of samples in the space of We, Wi.

        # The network:
        seed=0    # seed of random number generator to produce a random network, we can repeat the same
        N = 1000  # Number of neurons in a network.
        p = 0.02  # Sparseness of random network.
        Ne = 800  # Number of excitatory neurons.

        We_max = 50.0 # weight of excitatory synapses. Should be a positive float number.
        Wi_max = 100.0 # weight of inhibitory synapses. Should be a positive float number.


        # Times:
        T = 1024  # Simulation time in ms. It is better to be in a power of two, as FFT algorithm prefe
        dt = 0.001 # Time step of NeMo. Don't change it.

        freq = np.fft.rfftfreq(T,dt)  # Generate the values of frequencies according to T and dt.
```

```
# We simulate each network len(stim_list) times. Each time, we stimulate stim_list[i] neurons a
stim_list = [0, 1, 2, 4, 8, 16, 32, 64, 128, 256]

# Read the dataframe if it already exists.
# Create the data frame if it does not exist.
if os.path.isfile(filename):
    df=pd.read_csv(filename, index_col=0)
else:
    df = pd.DataFrame(columns=['N', 'p', 'Ne', 'We', 'Wi', 'Ns', 'seed', 'T',
                               'MaxRate', 'TMaxRate', 'MaxERate', 'TMaxERate', 'MaxIRate', 'TMaxIRa
                               'LastNonZero', 'fftDC', 'fftMax', 'FreqOfMax'])
```

1.2 Functions

In [3]:
```
def create_connections(N, p, seed=None):
    '''Returns a tuple of (source, target).

    N: number of neurons.
    p: sparsness of connections.
    seed: random seed.

    source and targets are numpy arrays of the same length. Together they define synapses.
    '''
    g = nx.generators.gnp_random_graph(N,p,seed=seed,directed=True)  # make a directed random g
    # makes the graph suitable for the neural simulation.
    src = np.array(g.edges())[:,0]  # source: the connection comes from this neurons.
    trg = np.array(g.edges())[:,1]  # target: the connection goes to this neurons.
    #nx.draw_circular(g)
    return src, trg


def create_network(N, Ne, source, target, We, Wi):
    '''set up and return a network to be simulated by NeMo.
    N: total number of neurons.
    Ne: exitatory neurons.
    source: source neurons of synapses.
    target: target neurons of synapses.
    We: weight of excitatory connections.
    Wi: weight of inhibitory connections.
    '''

    net = nemo.Network()
    iz = net.add_neuron_type('Izhikevich')

    # a, b, c, d, sigma, u, v
    net.add_neuron(iz, range(Ne), 0.02, 0.2, -65.0, 8.0, 0, -14, -70)    # Regular spiking at r
    net.add_neuron(iz, range(Ne,N), 0.10, 0.2, -65.0, 2.0, 0, -14, -70)  # Fast spiking at rest

    #source target delay weight plastic
    net.add_synapse(source[source<Ne], target[source<Ne], 1, We, False)
    net.add_synapse(source[source>=Ne], target[source>=Ne], 1, Wi, False)

    return net
```

```
def random(n):
    ''' Returns n non overlaping random points in a 2D plane.
    '''

    rnd = np.random.random((n,2))

    for i in range(10*n):
        idx = np.random.randint(n)
        old_pt = rnd[idx,:].copy()

        old_d = rnd - old_pt
        old_d = (old_d * old_d).sum(1)

        rnd[idx,:] = np.random.random(old_pt.shape)

        new_d = rnd - rnd[idx,:]
        new_d = (new_d * new_d).sum(1)

        # soft core potential so that points do not overlap.
        old_d = np.exp(-old_d*n*4).sum()
        new_d = np.exp(-new_d*n*4).sum()

        if new_d > old_d:
            rnd[idx,:] = old_pt.copy()
    return(rnd)

def max_dynamic_range(l):
    '''Find out the largest contiguous monotonic part of a list.
    '''
    l = np.array(l)
    l = np.insert(l,0,100000)
    l = np.append(l,-100000)
    d = np.diff(l)
    d = d[d!=0]  # remove zeros.
    negative = np.where(d<0)[0]
    return np.diff(negative).max()
```

1.3 The main loop

The main calculations are done here. We run it a couple of time to have more data points.

In [4]:
```
# generate the list of We and Wi pairs:
W_list = np.round(np.array([We_max,-Wi_max])*random(points),2)

rate = np.zeros(T,dtype=np.uint16)  # to store the firing rate at each time.
erate = np.zeros(T,dtype=np.uint16) # to store firing rate of excitatory neurons.
irate = np.zeros(T,dtype=np.uint16) # to store firing rate of inhibitory neurons.

start = time.clock()  # How much does it take?
source,target = create_connections(N, p, seed)
for We,Wi in W_list:
    net = create_network(N, Ne, source, target, We, Wi)
    sim = nemo.Simulation(net,conf)
    for Ns in stim_list:
```

```
            #rate.fill(0)
            rate[-1], erate[-1], irate[-1] = 0, 0, 0
            last_non_zero = 0
            sim.set_neuron_state(range(N),0,N*[-14]) # reset u
            sim.set_neuron_state(range(N),1,N*[-70]) # reset v
            sim.reset_timer()
            sim.step_f(range(Ns))
            for t in range(T):
                fired = np.array(sim.step_noinput())
                rate[t] = len(fired)
                erate[t] = (fired < Ne).sum()
                irate[t] = rate[t] - erate[t]
                last_non_zero = t if rate[t] else last_non_zero

            rate_fft = np.fft.rfft(rate) # calculate the real fourier transform
            rate_fft = (rate_fft * np.conj(rate_fft)).real # power spectra

            fftDC = rate_fft[0] * dt**2  # DC component
            rate_fft[0] = 0  # remove DC component.

            fftMax = rate_fft.max() * dt**2  # maximum power

            FreqOfMax = freq[rate_fft.argmax()]  # frequency of maximum power

            df.loc["{}_{:.3f}_{}_{:.2f}_{:.2f}_{}_{}_{}".format(N, p, Ne, We, Wi, Ns, seed, T)] = [
    print "Time passed in seconds:", time.clock() - start  # print the total elapsed time.
    df.to_csv(filename)  # save results.
```

```
Time passed in seconds: 134.891102
```

1.4 Calculate the dynamic range

```
In [7]: df.drop_duplicates()  # remove duplicates.
        df = df[df.N==1000][df.MaxERate < 0.9*df.Ne][df.MaxIRate < (df.N-df.Ne)] # keep trials with 100
        dfg = df.groupby(["N", "p", "Ne", "We", "Wi", "seed"], as_index=False)  # group data.
        dr = dfg.aggregate(max_dynamic_range) # Calculate Dynamic Ranges for each groups.
        med = dfg.aggregate(np.median)  # Calculate the median of each quantity. Usefull for filtering
        maxx = dfg.aggregate(np.max)  # Calculate the max of each quantity. Usefull for filtering data.
```

1.5 Plot the dynamic ranges:

```
In [11]: # Plot the dynamic range.
         # discard all the Hyper-sensitive networks, median of MaxRate > 1000.
         # discard all the non-sensitive networks, median of MaxRate = 0.
         # discard all the networks that fired for ever, median of LasNonZero firing rate is equal to 1
         dr[dr.p==p][dr.Ne==Ne][med.MaxRate<1000][med.MaxRate>0][med.LastNonZero<100].plot(kind="hexbin
                         C="MaxERate", vmin =1, vmax=10, gridsize=10, cmap=plt.cm.jet,  reduce_C_functi
```

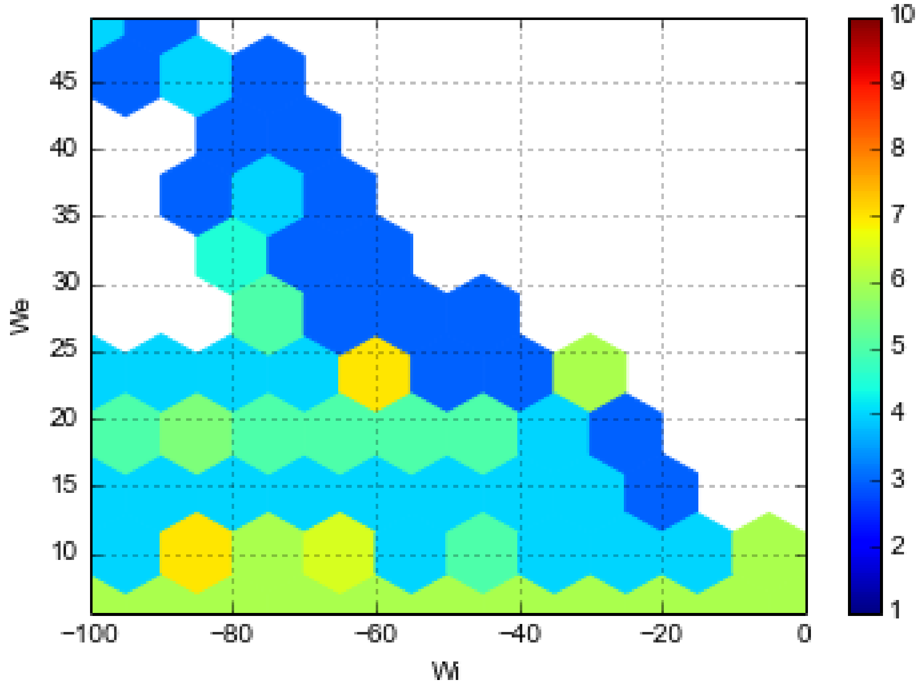

1.6 Oscillations

Here we plot the dominant frequency of each nework.

```
In [12]: df[df.p==p][df.Ne==Ne][df.MaxRate<1000][df.FreqOfMax>7][df.FreqOfMax<100].plot(kind="hexbin",x=
                             C="FreqOfMax", gridsize=10, cmap=plt.cm.jet, reduce_C_function=np.max);
```

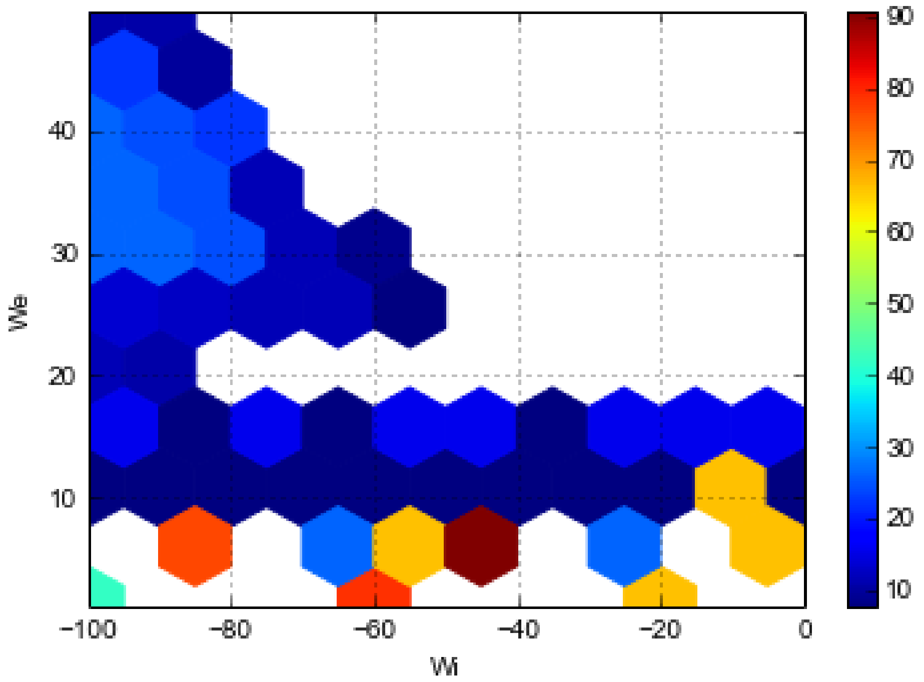

The rest is only a matter of dividing and conquering, organizing and making the graphs pretty. If we do so, the dynamic range become:

```
In [14]: Image(filename='dynamic-range.png')
```

Out[14]:

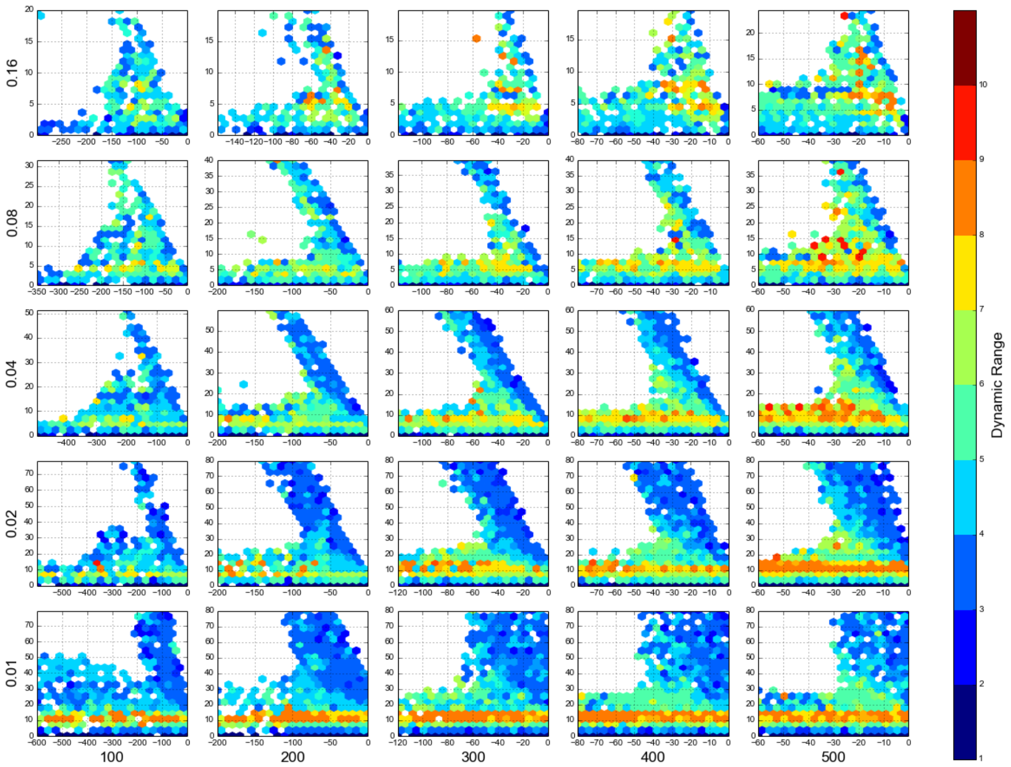

where numbers on the left are sparsnesses, and number below are the number of inhibitory neurons.

For oscillations, we have:

```
In [15]: Image(filename='oscillations.png')
```

Out[15]:

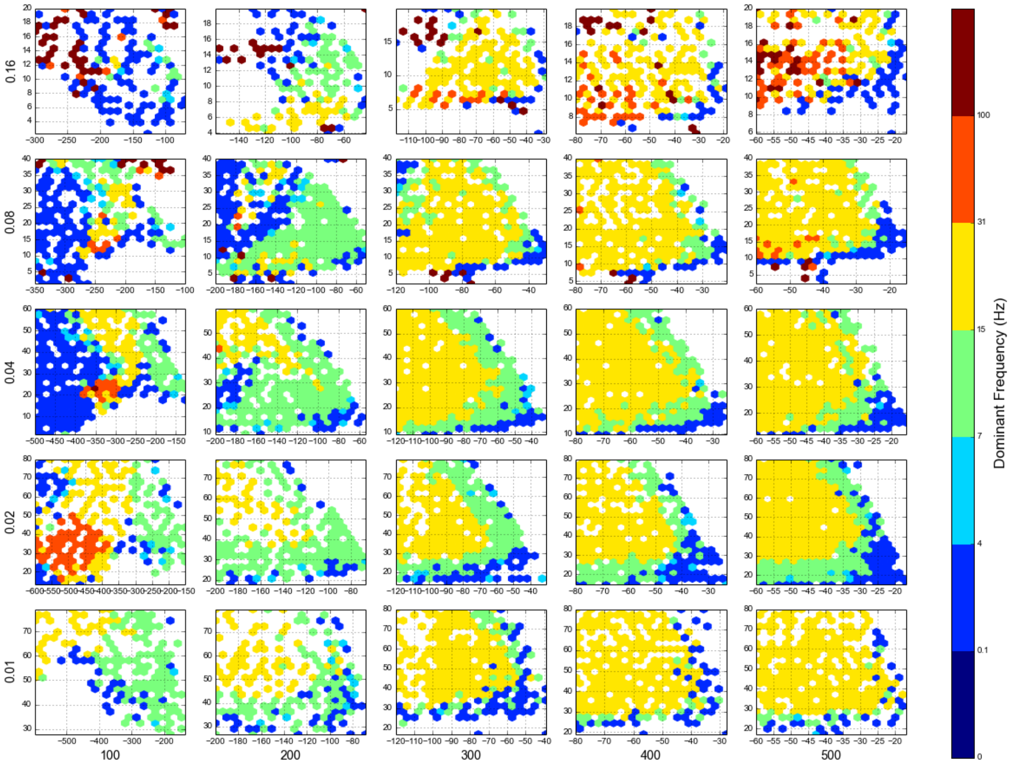

Here the colors are organized acording to brain waves:

- Delta: 0.1 – 4 Hz
- Theta: 4 – 7 Hz
- Alpha: 7 – 15 Hz
- Beta: 15 – 31 Hz
- Gamma: 31 – 100 Hz

2 Details of example networks

Now let zoom on some interesting cases.

2.1 Fast Response with a good Dynamic Range

Here we choose a set of parameters that generate a fast response with a decent dynamic range.

```
In [16]: max_dr = dr[maxx.LastNonZero<100][maxx.MaxERate<maxx.Ne].MaxERate.max()  # discard any thing l
         min_res_time = maxx[dr.MaxERate == max_dr][maxx.LastNonZero<100].TMaxERate.min()  # Find the s

         fastest_dr = dr[dr.MaxERate == max_dr][maxx.TMaxERate == min_res_time][maxx.LastNonZero<100]
```

```
We = fastest_dr.iloc[0].We
Wi = fastest_dr.iloc[0].Wi
p  = fastest_dr.iloc[0].p
N  = int(fastest_dr.iloc[0].N)
Ne = int(fastest_dr.iloc[0].Ne)
seed  = int(fastest_dr.iloc[0].seed)

print N, p, Ne, We, Wi, seed, max_dr
```

```
1000 0.02 800 9.97 -81.31 0 7.0
```

Now run the simulation for this parameters, and keep all the spikes.

```
In [17]: len(stim_list)

         rate = np.zeros((len(stim_list),T+1),dtype=np.uint16)  # to store the firing rate at each time
         erate = np.zeros((len(stim_list),T+1),dtype=np.uint16) # to store firing rate of excitatory ne
         irate = np.zeros((len(stim_list),T+1),dtype=np.uint16) # to store firing rate of inhibitory ne
         raster = [ [] for j in range(len(stim_list))]

         source,target = create_connections(N, p, seed)
         net = create_network(N, Ne, source, target, We, Wi)
         sim = nemo.Simulation(net,conf)
         i = 0
         for Ns in stim_list:
             raster[i]=[[],[]]
             rate[-1], erate[-1], irate[-1] = 0, 0, 0
             last_non_zero = 0
             sim.set_neuron_state(range(N),0,N*[-14]) # reset u
             sim.set_neuron_state(range(N),1,N*[-70]) # reset v
             sim.reset_timer()
             sim.step_f(range(Ns))
             for t in range(1,T+1):
                 fired=np.array(sim.step_noinput(), dtype=np.uint16)

                 raster[i][0].extend(len(fired)*[t])
                 raster[i][1].extend(list(fired))

                 rate[i,t] = len(fired)
                 erate[i,t] = (fired < Ne).sum()
                 irate[i,t] = rate[i,t] - erate[i,t]
                 last_non_zero = t if rate[i,t] else last_non_zero
             i+=1
```

Population rate vs time

```
In [18]: a = rate  # select the quantity that we want to see eg, rate, erate, irate
         Tmax = T-np.where(a[:,range(T-1,-1,-1)]>0)[1].min()
         Tmax = 50
         plt.plot(a[1:5,:Tmax].T,'--')
         plt.plot(a[5:,:Tmax].T)

         plt.xlim(-Tmax/4,Tmax)

         plt.legend(stim_list[1:])
```

```
plt.ylabel("Population firing rate")
plt.xlabel("Time")
#plt.savefig("dr-rates.eps")
```

Out[18]: <matplotlib.text.Text at 0x7fcc960d9c90>

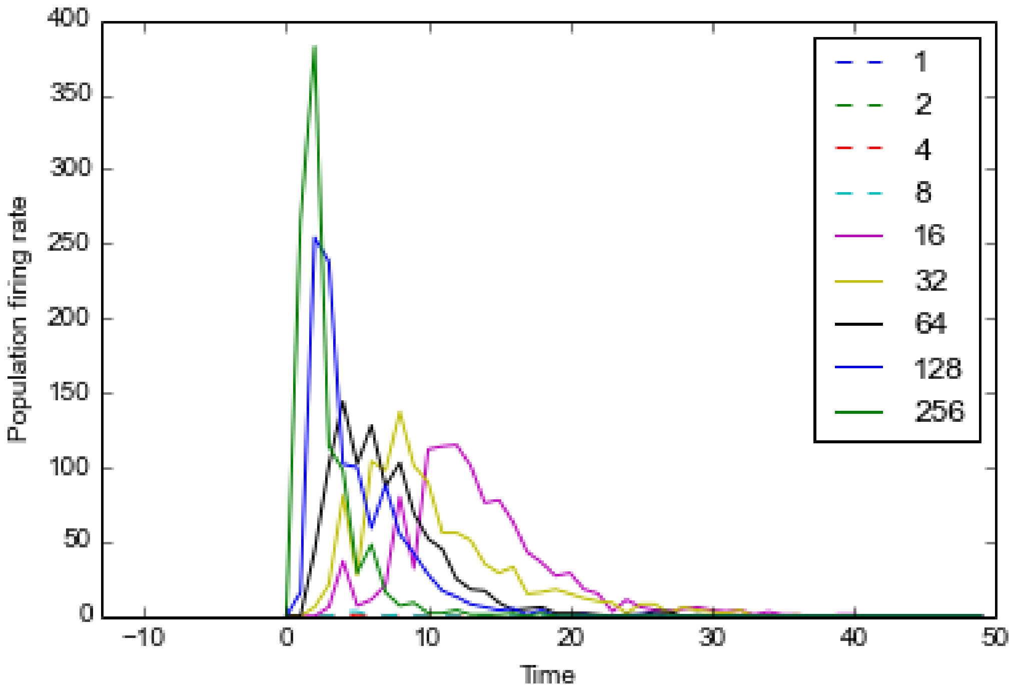

Raster plot

In [20]:
```
stim = 8  # make the graph for this stimuli.

plt.scatter(raster[stim][0], raster[stim][1], s=2, linewidths=0)
plt.xlim(-Tmax/4,Tmax)
plt.ylim(0,N)

plt.axhline(y=Ne, color='r')

plt.ylabel("Neuron number")
plt.xlabel("Time");
#plt.savefig("dr-raster.eps")
```

Out[20]: <matplotlib.text.Text at 0x7fcc961930d0>

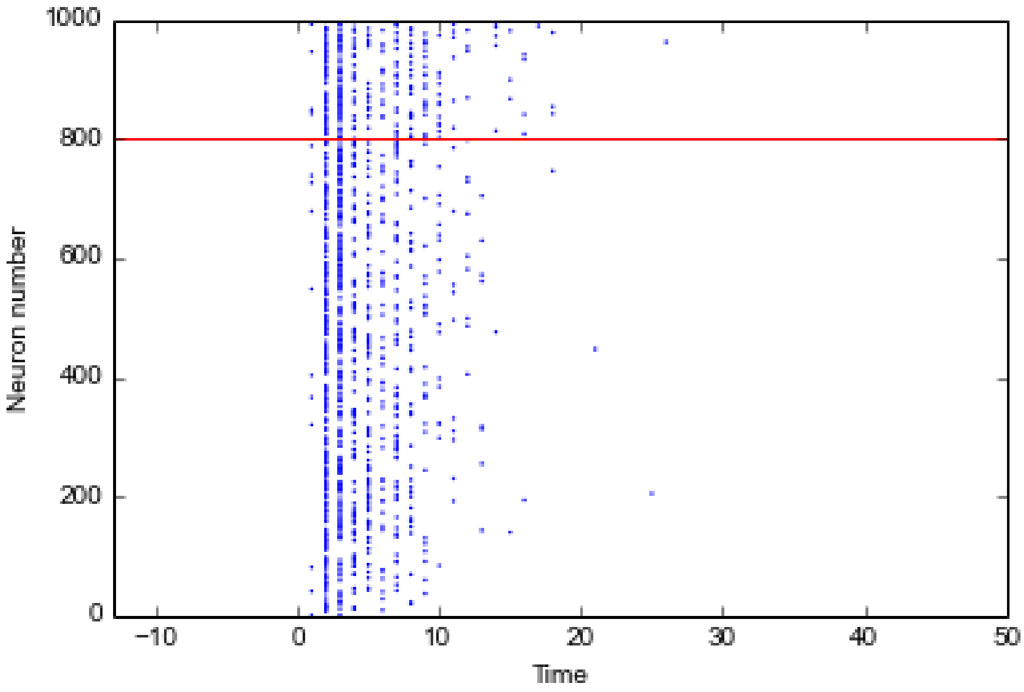

Neurons above the red line are inhibitory.

2.2 Oscillatory Networks

This time we choose network parameters that generate oscilations.

```
In [21]: # select trials with a dominant frequency between 0.1Hz to 15Hz
         # select networks that remain avtive until the end of simulation.
         sb = df[df.LastNonZero>1022][df.MaxERate<(0.5*df.Ne)][df.MaxIRate<0.9*(df.N-df.Ne)][df.FreqOfMa
         sf = sb[sb.fftMax == sb.fftMax.max()].iloc[0] # Among them, lets select one with strong power.
         We = sf.We
         Wi = sf.Wi
         N = int(sf.N)
         Ne = int(sf.Ne)
         p = sf.p
         seed = int(sf.seed)
         Ns = int(sf.Ns)
         stim_idx = np.where(stim_list == sf.Ns)[0][0]
         print N, p, Ne, We, Wi, seed, Ns, sf.FreqOfMax
```

```
1000 0.02 800 31.08 -98.52 0 1 13.671875
```

```
In [22]: rate = np.zeros((len(stim_list),T+1),dtype=np.uint16)  # to store the firing rate at each time
         erate = np.zeros((len(stim_list),T+1),dtype=np.uint16) # to store firing rate of excitatory ne
         irate = np.zeros((len(stim_list),T+1),dtype=np.uint16) # to store firing rate of inhibitory ne
         raster = [ [] for j in range(len(stim_list))]

         source,target = create_connections(N, p, seed)
         net = create_network(N, Ne, source, target, We, Wi)
         sim = nemo.Simulation(net,conf)
```

```
i = 0
for Ns in stim_list:
    raster[i]=[[],[]]
    rate[-1], erate[-1], irate[-1] = 0, 0, 0
    last_non_zero = 0
    sim.set_neuron_state(range(N),0,N*[-14]) # reset u
    sim.set_neuron_state(range(N),1,N*[-70]) # reset v
    sim.reset_timer()
    sim.step_f(range(Ns))
    for t in range(1,T+1):
        fired=np.array(sim.step_noinput(), dtype=np.uint16)

        raster[i][0].extend(len(fired)*[t])
        raster[i][1].extend(list(fired))

        rate[i,t] = len(fired)
        erate[i,t] = (fired < Ne).sum()
        irate[i,t] = rate[i,t] - erate[i,t]
        last_non_zero = t if rate[i,t] else last_non_zero
    i+=1
```

Population rate vs time

```
In [23]: a = erate  # select the quantity that we want to see eg, rate, erate, irate
         b = irate  # select the quantity that we want to see eg, rate, erate, irate
         stim = stim_idx

         Tmax = T-np.where(a[:,range(T-1,-1,-1)]>0)[1].min()

         plt.plot(a[stim,:Tmax].T)
         plt.plot(b[stim,:Tmax].T)
         plt.xlim(-64,Tmax)
         plt.legend(["Excitatory","Inhibitory"])

         plt.ylabel("Population firing rate")
         plt.xlabel("Time");
         plt.savefig("osci-rates.eps")
```

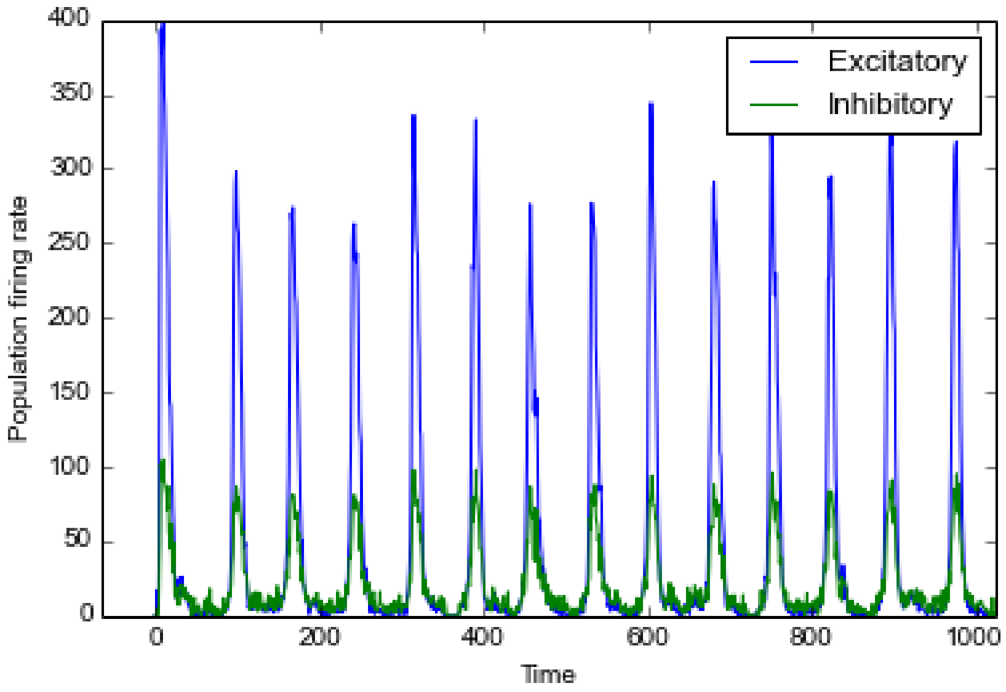

Raster plot

```
In [24]: stim = stim_idx  # make the graph for this stimuli.

         plt.scatter(raster[stim][0], raster[stim][1], s=2, linewidths=0)
         plt.xlim(-64,Tmax)
         plt.ylim(0,N)

         plt.axhline(y=Ne, color='r')

         plt.ylabel("Neuron number")
         plt.xlabel("Time");
         plt.savefig("osci-raster.eps")
```

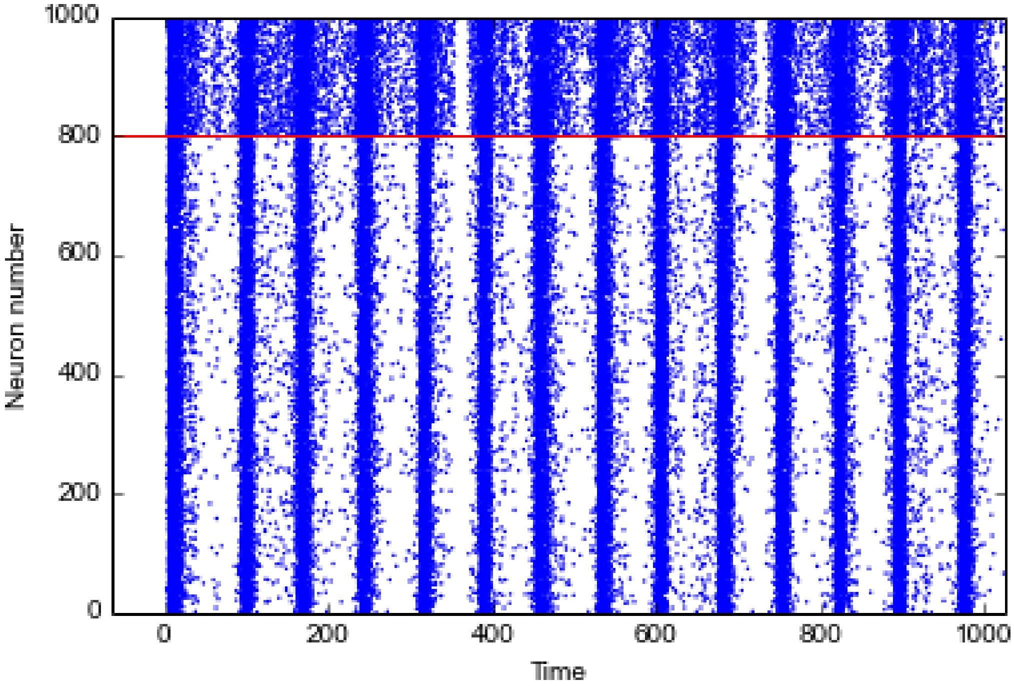

2.3 A pulse generates everlasting oscillations

The network was stimulated only by a pulse, there wasn't any current stimuli or spontaneous activity in the model.
